# Barriers to antigen detection and avoidance in chronic hypersensitivity pneumonitis in the United States

**DOI:** 10.1186/s12931-021-01817-6

**Published:** 2021-08-10

**Authors:** Kerri I. Aronson, Ronan O’Beirne, Fernando J. Martinez, Monika M. Safford

**Affiliations:** 1 Division of Pulmonary and Critical Care Medicine, Department of Medicine, Weill Cornell Medicine New York, 1305 York Avenue Y-1053, New York, NY 10021 USA; 2 Division of Continuing Medical Education, University of Alabama Birmingham, Alabama, USA; 3 Division of General Internal Medicine, Department of Medicine, Weill Cornell Medicine, New York, NY USA

## Abstract

**Background:**

Chronic hypersensitivity pneumonitis (CHP) is an interstitial lung disease (ILD) caused by long term exposure to an offending antigen. Antigen avoidance is associated with improved outcomes. We are unable to identify the antigen source in approximately half of patients. When an antigen is successfully identified, patients have difficulty with avoidance.

**Methods:**

We conducted three structured group discussions with US based ILD specialists utilizing the nominal group technique (NGT). Participants listed barriers to antigen detection and avoidance in CHP. Each participant ranked what they perceived to be the top three barriers in the list in terms of importance. The master list of barriers was consolidated across the three groups into themes that were prioritized based on receiving the highest rankings by participants.

**Results:**

Twenty-five physicians participated; 56% had experience caring for CHP patients for ≥ 16 years. Sixty barriers to antigen detection were categorized into seven themes of which the top three were: 1. unclear significance of identified exposures; 2. gaps in clinical knowledge and testing capabilities; 3. there are many unknown and undiscovered antigens. Twenty-eight barriers to antigen avoidance were categorized into five themes of which the top three were: 1. patient limitations, financial barriers and lack of resources; 2. individual patient beliefs, emotions and attachments to the antigen source; and 3. gaps in clinical knowledge and testing capabilities.

**Conclusions:**

This study uncovered challenges at the individual patient, organizational, and societal levels and ranked them in terms of level of importance. These findings provide information to guide development and validation of multidisciplinary support and interventions geared towards antigen identification and avoidance in CHP.

**Supplementary Information:**

The online version contains supplementary material available at 10.1186/s12931-021-01817-6.

## Background

Chronic hypersensitivity pneumonitis (CHP) is an interstitial lung disease (ILD) in which injury to the lung is caused by an immune reaction in a sensitized person to an inhaled environmental antigen. In contrast to other forms of ILD, early recognition of CHP may result in halting progression if the offending antigen is both successfully identified and avoided. Unfortunately, even with a confident diagnosis of CHP, we are only able to successfully identify the antigen in around half of our patients [1]. The ability to identify an antigen is important in the diagnostic process, helping to tease out cases of CHP vs other ILD such as idiopathic pulmonary fibrosis (IPF) when other data is less informative [2]. The inability to identify an exposure has been cited as one of the biggest challenges in making the diagnosis of CHP, and is regarded as highly valuable in the diagnostic process [3, 4].

Aside from diagnostic utility, identification of an antigen affects patient outcomes. There are limited data on the efficacy of current therapies used for CHP, and none have shown mortality benefit [5–8]. In contrast, the ability to identify an antigen, is a significant predictor of survival [1]. Additionally, in our prior work, we learned that antigen detection and avoidance impacts multiple components of quality of life including patients’ daily activities, employment, finances, and their home environment [9].

Over 200 antigens have been identified as potential contributors to CHP. These can exist in a person’s home, workplace, or recreational environment [10, 11]. Unfortunately, there is no standardized method to identify an antigen [12]. Serum precipitin testing and specific inhalation challenges have been employed but these tests lack sensitivity and specificity for individual patients and regions where individual exposures may differ [13, 14].

If we do successfully identify the culprit antigen, avoidance often requires asking patients to modify or avoid a certain environment, or to rid themselves of a personal belonging or pet [15]. While we believe this may be an effective solution for disease control, it ultimately may not be possible to avoid the antigen source. This leaves us with questions about how best to proceed with CHP management and provide further recommendations to patients.

Among the studies performed on patients with CHP, none to our knowledge have systematically highlighted barriers to antigen detection and avoidance. Given the implication that identifying an antigen has on disease diagnosis, treatment and prognosis, it is highly important that significant attention is paid to the most common barriers to achieving this. This knowledge will provide us with concrete targets for research and clinical care. The aim of this study was to elicit the perspectives of expert ILD physicians and to identify and prioritize the barriers to antigen identification and avoidance in CHP. We collected the data using the nominal group technique (NGT) which allows for the systematic collection and prioritization of challenges and barriers in clinical practice [16, 17].

## Methods

### Study participants

Pulmonologists from academic pulmonary fibrosis care centers of excellence across the United States with clinical expertise in ILD were invited to participate in the study. To be included, providers needed 5 or more years of experience treating patients with CHP (including fellowship training years). Pulmonologists without specialization in ILD, practicing less than 5 years, and trainees were excluded. A total of 155 practitioners were invited to participate in one of three online NGT sessions. The maximum number of participants allowed to enroll per group was 12. The study was approved by the Weill Cornell Medicine Institutional Review Board (IRB Protocol #1810019697). Informed consent was waived for the purposes of this study.

### Data collection

The NGT is a well validated and effective method for identification of challenges and barriers in medical practice and priority setting amongst a group of stakeholders [18, 19]. It is a structured group discussion led by a moderator that allows for collection of qualitative data. This method helps to identify and prioritize problems, allowing the establishment of an appropriate research agenda to devise targeted solutions [20].

A total of three NGT sessions were conducted in August 2019 using a virtual software tool. Participants dialed a toll-free number and simultaneously logged onto a secure website designed to support NGT sessions. The sessions were audio-recorded. The sessions were moderated by two investigators (MMS and KIA) who are both experienced group moderators. Participants were asked to share their perspectives on the topic by answering two distinct questions related to antigen identification and separately, antigen avoidance in CHP. The questions were pilot tested with three pulmonologists prior to the sessions.

### Question 1:

“Picture yourself taking care of your patients with chronic hypersensitivity pneumonitis. What are some of the challenges you face when helping your patients to identify the antigen that is causing their lung disease?”

### Question 2:

“Now picture that you have successfully identified the antigen. What are some of the challenges your patients face when trying to avoid the antigen?”

**Fig. 1:**
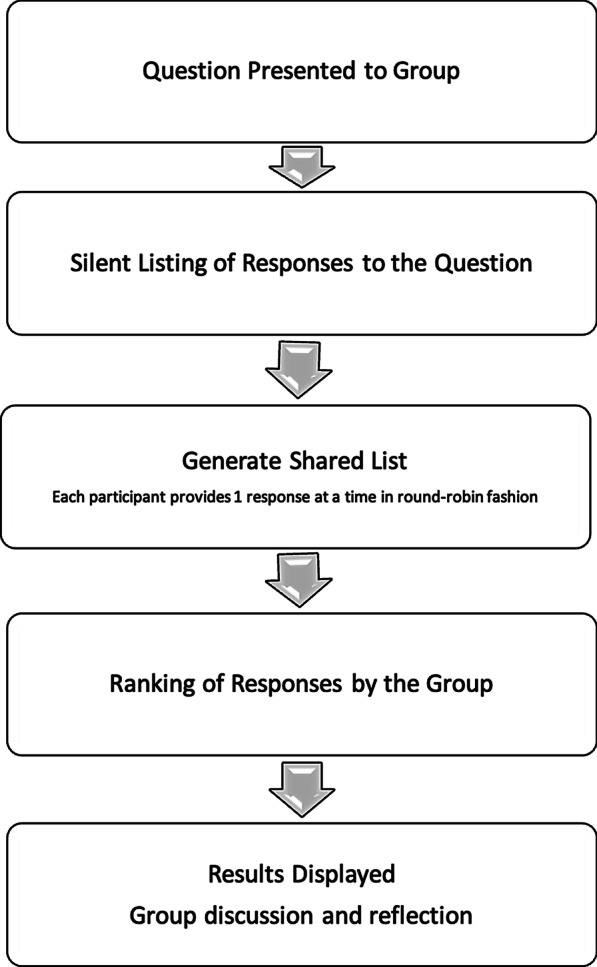
Diagram outlining stages of the nominal group technique

Each question was allocated 45 min. After a brief introduction, the first question was displayed on the website and participants were given 5 min during which they silently listed their responses to the question. Next, participants were asked to share a single barrier, one-by-one in round-robin fashion. This process continued until all participants had no new barriers to share with the group. The process that details the steps of the nominal group session is shown in Fig. 1. All barriers were entered verbatim into the software in real time by a research assistant. Once all barriers were recorded for each question, participants were able to view the list of barriers. Participants were given the opportunity to correct or clarify the barriers to ensure that they were documented correctly by the study team and understood by all participants. There was also a brief opportunity for participants to discuss these barriers in more detail prior to ranking. We then moved on to ranking the barriers. In this phase, each participant was asked to select the most important barrier, in their opinion; they were then asked to select the second most important barrier, and then the third most important barrier. The NGT software then generated a list of the most highly prioritized barriers for that group of participants. The prioritized list was then reviewed by the group and briefly discussed.

### Data analysis

Once the sessions were completed, the barriers from all groups were combined into two master lists, with antigen detection question and antigen avoidance treated separately. Three members of the research team each independently reviewed the two master lists and identified similar barriers identified between the three groups. The three reviewers then discussed their findings and came to consensus on combining these similar barriers into a single barrier for each master list. The barriers on the master lists were then organized into broader themes. We noted the number of barriers included in each theme, and the number of nominal groups that discussed the theme.

To summarize the groups’ prioritized barriers and themes, we assessed the total number of votes and points for each barrier and, separately, each theme. Each participant had a total of 3 prioritized votes; the most important barrier was assigned 3 points, the second most important barrier was assigned 2 points, and the third most barrier was assigned 1 point. Barriers that received no priority votes received a score of zero. With 25 participants, across the three groups and three votes and 6 points per participant, there were a total of 75 votes and 150 points.

## Results

### Participant characteristics

A total of 32 practitioners registered for the study. Due to last minute scheduling conflicts, 25 (78%) of practitioners participated in the group sessions. More than half of the participants had experience caring for CHP patients for 16 years or more and the median percentage of patients with CHP in the participants’ practices was 14.3%. A majority of participants were from the Northeast, Southeast, and Midwest regions of the United States (Table 1).

**Table 1:** Characteristics of the ILD physicians who participated in the nominal groups on barriers to antigen identification and avoidance in CHP

Participant characteristics	Distribution (n = 25)
Years caring for CHP Patients	
0–5	4
6–10	6
11–15	1
16–20	8
20+	6
Percentage of CHP patients in the provider’s practice	14.3% (3–35%)
US Region of Practice [21]	
Northeastern United States	7
Southeastern United States	8
Midwestern United States	8
Southwestern United States	1
Western United States	1
Session Participation (# of participants)	
Session 1	6
Session 2	10
Session 3	9

CHP, Chronic Hypersensitivity Pneumonitis; ILD, Interstitial Lung Disease

US, United States

### Identified themes

Responses to question 1 resulted in a total of 60 unique barriers to antigen detection in CHP. These barriers were classified into 7 major themes: 1. unclear significance of identified exposures; 2. gaps in clinical knowledge and testing capabilities; 3. there are still many unknown and undiscovered antigens; 4. problems with obtaining an accurate and comprehensive exposure history; 5. patient limitations, financial barriers and lack of resources; 6. individual patient beliefs, emotions and attachments to antigen source; 7. problems with environmental inspections and testing. All of these 7 themes were discussed in all 3 groups. The majority of barriers were included in three themes: gaps in clinical knowledge and testing capabilities; problems with obtaining an accurate and comprehensive exposure history; and patient limitations, financial barriers and lack of resources (Table 2).

**Table 2:** Identified themes for question 1: barriers to antigen identification in CHP

Theme	Number of barriers in the theme (n = 60)
Unclear significance of identified exposures	9
Gaps in clinical knowledge and testing capabilities	12
There are still many unknown and undiscovered antigens	5
Problems with obtaining an accurate and comprehensive exposure history	12
Patient limitations, financial barriers and lack of resources	11
Individual patient beliefs, emotions, and attachments to antigen source	5
Problems with environmental inspections and testing	6

All themes were discussed in all 3 nominal groups

Responses to question 2 resulted in a total of 28 unique barriers to antigen avoidance in CHP. These barriers were classified into 5 major themes: 1. individual patient beliefs, emotions and attachments to antigen; 2. effects on employment; 3. patient limitations, financial barriers and lack of resources; 4. gaps in clinical knowledge and testing capabilities; 5. limitations with environmental remediation (Table 3). All of these 5 themes were discussed in all 3 groups. The majority of barriers were included in three themes: individual patient beliefs, emotions, and attachments to antigen; limitations with environmental remediation; and gaps in clinical knowledge and testing capabilities.

**Table 3:** Identified themes for question 2: barriers to antigen avoidance in CHP

Theme	Number of barriers in the theme (n = 28)
Individual patient beliefs, emotions, and attachments to antigen source	8
Effects on employment	2
Patient limitations, financial barriers and lack of resources	6
Gaps in clinical knowledge and testing capabilities	5
Limitations with environmental remediation	7

All themes were discussed in all 3 nominal groups

A full list of the individual barriers identified by participants are available in Additional file 1: Tables S1 and S2.

### Ranking

**Fig. 2:**
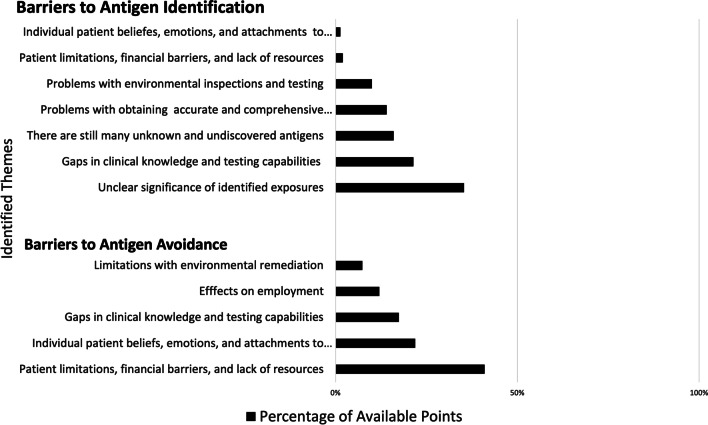
Barriers to antigen identification and avoidance, themes ranked by percentage of points. A total of seven themes were identified for barriers to antigen identification. A total of 5 themes were identified for barriers to antigen avoidance. The percentage of available points for barriers to antigen identification and antigen avoidance were calculated separately with a total of 150 points available for each

For question 1 a total of 13 barriers were prioritized, belonging to 5 out of the 7 identified themes. The top three ranked themes were: there are still many unknown and undiscovered antigens; unclear significance of identified exposures; and gaps in clinical knowledge and testing capabilities (Table 4). Items within the theme “unclear significance of identified exposures” received the most points with 35% of the available points across the three groups. This was followed second by barriers within the theme “gaps in clinical knowledge and testing capabilities” which received 22% of the available points, and third by barriers in the theme “there are still many unknown and undiscovered antigens” which received 16% of the available points across the three groups (Fig. 2).

**Table 4:** Individual statements (barriers) to antigen identification that were ranked

Theme	Statement	Groups that ranked the statement (n = 3)
There are still many unknown and undiscovered antigens	Half of the time no antigen is identifiable	1,2
	Ubiquitous nature for potential exposures e.g., mold in a significant number of ILD patients	3
Unclear significance of identified exposures	Patients may have many potential exposures, difficult to know which are relevant or may be causing the disease	1,2,3
	There is a question of temporal relationship of the identified exposure	1,2
	Unclear if identified exposure is significant or intense enough to cause disease	2,3
	Difficulty in quantifying level or significance of exposure	1
	No known test that confirms that an antigen identified is actually causing the disease	1
Gaps in clinical knowledge and testing capabilities	The commercially available hypersensitivity panel is neither sensitive no specific	1,2,3
Problems with obtaining an accurate and comprehensive exposure history	There is no comprehensive user and time friendly evidence-based questionnaire to ask about exposures in the clinic	2,3
	Obtaining complete occupational and recreational exposure	1
Problems with environmental inspections and testing	Lack of professional resources to look for antigens in the home or workplace	3
	Cost and availability of environmental sampling and relevance to CHP	2

This table displays all of the identified barriers that were ranked by any group, the group who ranked the barrier, and the key theme the barrier belongs to. These statements are not listed in order of rank. ILD, Interstitial Lung Disease, CHP, Chronic Hypersensitivity Pneumonitis

For question 2 a total of 8 barriers were prioritized across all 5 of the identified themes. The top ranked barrier in each of the 3 groups belonged to the “patient limitations, financial barriers and lack of resources” theme and was ranked number one in all groups (Table 5). This theme received the highest number of points (35%) across the three groups. This was followed by the theme “individual patient beliefs, emotions, and attachments to antigen” which received 23% of the available points and third by barriers in the theme “gaps in clinical knowledge and testing capabilities” which received 17% of the available points across the three groups (Fig. 2).

**Table 5:** Individual statements (barriers) to antigen avoidance that were ranked

Theme	Statement	Groups that ranked the statement
Patient limitations, financial barriers and lack of resources	Cost-including remediation of a home, moving to a different home, or changing occupation or livelihood	1,2,3
	Removal or avoidance may not be under the patient’s control	2
Individual patient beliefs, emotions, and attachments to antigen source	The patient has a hobby, passion, or emotional connection to the exposure, or something associated with the exposure, may affect quality of life	2
	When there is lack of clinical improvement despite antigen avoidance, makes it hard to convince the patient to avoid	3
Effects on employment	Exposure at workplace, employer may be unable or unwilling to remediate, or livelihood is tied to the exposure and the patient is unable to leave their job	1
	Work exposure may lead to job switches, vocational rehab, or disability to avoid the antigen	2
Gaps in Clinical knowledge and testing capabilities	Lack of guidelines on what is acceptable or enough antigen avoidance	3
Limitations with environmental remediation	Total or zero avoidance may not be possible	1

This table displays all barriers that were ranked by any group, the group who ranked the barrier, and the key theme the barrier belongs to. These statements are not listed in order of rank. ILD, Interstitial Lung Disease; CHP, Chronic Hypersensitivity Pneumonitis

Barriers to antigen identification and avoidance shared three themes: gaps in clinical knowledge and testing capabilities; patient limitations, financial barriers, and lack of resources; and individual patient beliefs, emotions, and attachments to antigen source.

## Discussion

Our study utilized the nominal group technique to identify and prioritize barriers to antigen detection and avoidance in CHP from the perspective of expert physicians who treat these patients. To our knowledge this is the first study aimed at systematically compiling and prioritizing these challenges. While there has been some headway on therapy for fibrotic CHP and other potential therapeutic targets on the horizon, antigen detection and avoidance remain a vital components of the diagnosis and management of CHP [4, 5, 22].

Ability to confirm the significance of identified exposures was highly prioritized by this group of ILD providers as a major barrier to antigen identification. This challenge is multifaceted and complex. It includes determining the amount of the exposure elicited in the history that is necessary to confirm causality, establishing a temporal relationship of the exposure to onset of disease, and correctly isolating the relevant exposure when several are identified. Addressing this challenge requires a multipronged approach focused at both the individual and broader systems levels. At the organizational level with respect to the healthcare system, research aimed at understanding relationships between the pathology of CHP and the burden of exposure is needed [23]. There are small conflicting studies reporting on the degree of avian exposure and how it relates to progression of disease [24–26]. Larger case control and prospective longitudinal studies aimed at quantifying the significance of a variety of exposures as they relate to symptom onset and objective clinical parameters will be of value in determining the necessary duration and timing of exposure that is required for an identified antigen to be considered the cause of CHP. It can be daunting to consider the sheer number of possible exposures. A centralized resource of potential antigens may be useful for updates in real time as more exposures are identified, and several pioneers in our field have begun to build such repositories [27]. Future validation of this type of method is warranted to understand if it leads to improved antigen detection and understanding of less commonly considered exposures that may be relevant.

Gaps in clinical knowledge and testing capabilities was ranked highly related to both antigen identification and avoidance. CHP may initially be misdiagnosed as another class of ILD, for example, IPF [28]. The recent development of a multi-society clinical practice guideline for the diagnosis of CHP is a positive step in the field as suspicion for CHP will determine the importance placed on a potential exposure [4]. There is a need for improvement in serum precipitin test, inhalational challenge tests, antigen avoidance tests and defining the role of bronchoalveolar lavage (BAL) for adjunctive information related to antigen detection [29–35]. Preliminary work has been performed with attempts to improve individualized exposure assessments in conjuncture with serum precipitin testing, however as this group of ILD experts pointed out, the lack of test sensitivity remains a continued problem [12]. We may also consider that this refinement of testing be coupled with individualizing environmental exposure assessments by the patient and the environment/community in which they reside [14, 36, 37]. There is now interest in using molecular signatures to help us to distinguish CHP from other fibrotic interstitial lung disease. The availability of this type of information may bring increased confidence that the identified exposures are truly causative of disease in a subset of patients who carry these distinct molecular signatures [38].

Challenges with obtaining accurate and comprehensive exposure histories from patients were commonly mentioned by this group of experts. Several groups have developed exposure assessment tools with ongoing efforts to validate comprehensive exposure surveys [39, 40]. Extending this validation to real-world settings will be important in the quest to collect a comprehensive history in a time-efficient and user-friendly manner. The group of experts in our study also brought to light the difficulty that many pulmonologists have with occupational exposure assessments. Enhancing provider education about occupational exposures, and improving access to national and local guideline resources and exposure databases through well respected and established occupational health organizations (e.g. National Institute for Occupational Safety and Health (NIOSH) guidelines for Health Hazard Evaluations (HHEs) and personal protective equipment (PPE) at the workplace [41, 42]). Could potentially address this issue. These agencies often deal with high levels of exposure that cause well established occupational lung diseases aside from HP. Increased communication and research partnerships between environmental and occupational medicine and pulmonary medicine to increase awareness and leverage the expertise of both groups would be of value.

Problems with environmental inspections and testing related to antigen identification received a fair number of points during the ranking process. Requirements for professional environmental inspection services need validation and increased transparency so that patients and providers are not left wondering if a sufficient inspection was performed. Conversely, patients and providers should have a level of confidence that an environmental inspection did not lead to identifying an insignificant exposure which led to more cost on behalf of the patient. In addition to standardizing inspection, research demonstrating that inspections improve outcomes will provide evidence for policy change around insurance coverage for exposure assessments in the home and workplace [43]. Currently such inspections are paid for out of pocket and are beyond the financial reach of many patients.

As we confirmed in this study, identifying an antigen is only part of the journey to a successful outcome in CHP. Identifying an antigen loses utility if the patient cannot successfully avoid it. Based upon the findings in this study, (and that notably three of the identified themes overlapped between the two questions) we propose that the approach to improving avoidance is similar, and in many ways overlapping with the approach to antigen identification. This group of ILD specialists unanimously ranked “patients’ limitations, financial barriers, and lack of resources” as the top challenge to antigen avoidance. Interestingly, this theme was identified from both questions, but unanimously ranked as the top challenge to antigen avoidance. While not ranked highly as a barrier for antigen identification by percentage of available points, the actual number of responses included in that theme (that were not subsequently ranked) was high. This was also true for “individual patient beliefs, emotions, and attachments to antigen”, (a theme describing barriers such as patients’ own beliefs about the diagnosis and their emotional attachments to sources of antigen such as pets or hobbies) which was uncovered as a theme from both questions, but only ranked highly when related to antigen avoidance. The interesting and potentially important difference here suggests that antigen identification is thought of as a process more reliant on the actions of the clinician and medical system while the act of avoidance is more reliant on the individual patient. As we have found in our prior work, there can be substantial socioeconomic and psychological impact of antigen identification and avoidance on patients living with CHP [9]. This study highlights the need to prioritize ways to address these barriers when exploring exposures and making recommendations for avoidance. This shines a light on the need for a broader multidisciplinary approach that does not place the actions of the clinician, medical system, community, and patient into silos but instead provides an integrated approach to addressing the barriers.

A similar approach has been successfully adopted in asthma management. Community-based environmental approaches are known to improve asthma outcomes by focusing on barriers that patients face in the community and linking clinical care with the individual patient environment [44–46]. We propose that we should strive to achieve a similar multidisciplinary care model for our patients with CHP, but to achieve this goal will require further resources and research. Improving allocation of resources to patients and their families and increased multidisciplinary efforts within the medical system-such as improved access to medical care and avenues of support that help to address knowledge-gaps-may positively affect patient biases and beliefs about antigen identification and avoidance.

This study had limitations. The results represent the perspectives of a group of ILD providers in the United States and does not take into account international perspectives where availability of testing, environmental and occupational standards, and health insurance coverage may differ. While the group was relatively small, all participants were ILD specialists, who care for more CHP patients than the general pulmonologist. While there was representation from all regions of the United States, there was more representation from the Northeast, Midwest and Southeast which may limit generalizability. A future survey of a larger group of pulmonologists would be a future direction of this research.

## Conclusion

Several experts have proposed a “call to action” related to gearing up resources and time to further research for CHP diagnosis and management [47–49]. While we continue to improve time to diagnosis, investigate genetic footprints and develop targeted therapies, we cannot forget about the large and “unidentified” elephant in the room: the antigen source. This is the first study to fully explore, identify, and prioritize the challenges to antigen discovery and avoidance in CHP. Based upon the results, we propose future research efforts and resource allocation devoted to validating multidisciplinary approaches to antigen detection and avoidance with focus on the highly prioritized barriers uncovered here. This will require targeting interventions at the individual patient level as well as allocating resources at the community and public policy level [50].

## Supplementary Information

**Additional file 1: Table S1.** List of all Individual Barriers to Antigen Identification. **Table S2.** List of all Individual Barriers to Antigen Avoidance.

## Data Availability

The datasets used and/or analysed during the current study are available from the corresponding author upon reasonable request.

## Abbreviations

BAL: Bronchoalveolar Lavage
CHP: Chronic Hypersensitivity Pneumonitis
ILD: Interstitial Lung Disease
NGT: Nominal Group Technique
IPF: Idiopathic Pulmonary Fibrosis
