# Supplementary material for: Barriers to antigen detection and avoidance in chronic hypersensitivity pneumonitis in the United States
Source: Respir Res. 2021 Aug 10;22:225. doi: 10.1186/s12931-021-01817-6 (PMC8353836; doi:10.1186/s12931-021-01817-6)
Supplement: Supplementary file 1 — Additional file 1: Table S1. List of all Individual Barriers to Antigen Identification. Table S2. List of all Individual Barriers to Antigen Avoidance. [file 12931_2021_1817_MOESM1_ESM.docx]

**Table S1: List of all Individual Barriers to Antigen Identification**

| All barriers discussed in more than 1 group | | | | | |
| --- | --- | --- | --- | --- | --- |
|  | **Groups barrier was discussed in** | **Theme barrier was assigned to** | **Barrier** | **Number of Points**  **(n=150)** | **Number of Votes**  **(n=75)** |
| 1 | 1,2 | There are many unknown and undiscovered antigens | Half of the time no antigen is identifiable. | 18 | 7 |
| 2 | 1,2,3 | Unclear Significance of Identified Exposures | Patients may have many potential exposures and it is difficult to know which are relevant or may be causing the disease. | 18 | 9 |
| 3 | 2,3 | Unclear Significance of Identified Exposures | It is unclear if the identified exposure is significant or intense enough to cause disease. | 9 | 4 |
| 4 | 1,2 | Unclear Significance of Identified Exposures | There is a question of the temporal relationship of the identified exposure. | 10 | 5 |
| 5 | 1,2,3 | Gaps in clinical knowledge and testing capabilities | The commercially available hypersensitivity panel is neither sensitive nor specific. | 18 | 10 |
| 6 | 2,3 | Problems with obtaining accurate and comprehensive exposure history | There is no comprehensive user and time friendly evidence based questionnaire to ask about exposures in the clinic. | 13 | 7 |
| 7 | 2,3 | Gaps in clinical knowledge and testing capabilities | Physicians’ lack understanding about occupational exposures and have difficulty taking this history. | 4 | 2 |
| 8 | 1,2,3 | Problems with environmental inspections and testing | It is hard to know what inspection company to use, as there are variations in credentials, knowledge, and experience. | 0 | 0 |
| 9 | 1,3 | Problems with obtaining accurate and comprehensive exposure history | There may be language barriers when taking a history. | 0 | 0 |
| Barriers discussed only in group 1 | | | | | |
|  | **Groups barrier was discussed in** | **Theme barrier was assigned to** | **Barrier** | **Number of Points**  **(n=150)** | **Number of Votes**  **(n=75)** |
| 10 | 1 | Unclear Significance of Identified Exposures | There is difficulty in quantifying the level or significance of an exposure. | 10 | 4 |
| 11 | 1 | Unclear Significance of Identified Exposures | There is no known test that confirms that the antigen identified is actually causing the disease. | 3 | 2 |
| 12 | 1 | Problems with obtaining accurate and comprehensive exposure history | There is difficulty obtaining a complete occupational and recreational exposure history. | 3 | 1 |
| 13 | 1 | Patient limitations, financial barriers, and lack of resources | For people who live in public housing, there is an inability to alter their home environment. | 2 | 1 |
| 14 | 1 | Gaps in clinical knowledge and testing capabilities | The lack of certainty of a diagnosis of hypersensitivity pneumonitis. | 1 | 1 |
| 15 | 1 | Problems with obtaining accurate and comprehensive exposure history | We rarely remember to ask about down products even though these are major source of exposure. Patients may not know which things in their home contain down. | 1 | 1 |
| 16 | 1 | Gaps in clinical knowledge and testing capabilities | There is confusion about humidifiers and de-humidifiers. | 0 | 0 |
| 17 | 1 | Patient limitations, financial barriers, and lack of resources | The high cost of home mold inspection. | 0 | 0 |
| 18 | 1 | Problems with obtaining accurate and comprehensive exposure history | Physicians not using plain language to ask the questions. | 0 | 0 |
| 19 | 1 | Individual patient beliefs, emotions, and attachments to antigen source | A patient who doesn't believe that they have significant mold exposure in their environment. | 0 | 0 |
| 20 | 1 | Patient limitations, financial barriers, and lack of resources | A patient who lacks resources for renovation or for moving away. | 0 | 0 |
| 21 | 1 | Individual patient beliefs, emotions, and attachments to antigen source | A patient with a bird who denies exposure. | 0 | 0 |
| 22 | 1 | Unclear Significance of Identified Exposures | A patient whose disease progresses despite removal of the antigen. | 0 | 0 |
| 23 | 1 | Patient limitations, financial barriers, and lack of resources | Patients with this disease may be physically limited and they reply “no” about their environment because they have the inability to access areas of the home that may be affected. | 0 | 0 |
| 24 | 1 | There are many unknown and undiscovered antigens | There are so many possibilities and so little time to ask, unknown causes, or causes that are known but we are uncertain of. | 0 | 0 |
| 25 | 1 | Gaps in clinical knowledge and testing capabilities | There is a perception that all mold that is dangerous is dark in color which is incorrect. | 0 | 0 |
| 26 | 1 | Problems with obtaining accurate and comprehensive exposure history | When taking a history, sometimes we may not ask the questions specifically enough. Especially related to exposure in the home. | 0 | 0 |
| Barriers discussed only in group 2 | | | | | |
|  | **Groups barrier was discussed in** | **Theme barrier was assigned to** | **Barrier** | **Number of Points**  **(n=150)** | **Number of Votes**  **(n=75)** |
| 27 | 2 | Problems with environmental inspections and testing | Cost and availability of environmental sampling and relevance to CHP. | 7 | 3 |
| 28 | 2 | Gaps in clinical knowledge and testing capabilities | A majority of tests come back negative and we are uncertain what to do next. | 6 | 2 |
| 29 | 2 | Individual patient beliefs, emotions, and attachments to antigen source | Patients themselves may be reluctant to explore certain potential exposures. | 1 | 1 |
| 30 | 2 | Patient limitations, financial barriers, and lack of resources | The cost of home inspection is often thousands of dollars and beyond the budget of a lot of patients. | 1 | 1 |
| 31 | 2 | Individual patient beliefs, emotions, and attachments to antigen source | This is often a probabilistic discussion and patients want more black and white – the patient acceptance of this diagnosis can be challenging | 1 | 1 |
| 32 | 2 | Problems with obtaining accurate and comprehensive exposure history | The ability to identify potential exposures at the work environment | 0 | 0 |
| 33 | 2 | Problems with environmental inspections and testing | Unavailability of industrial hygienists to go out to the home, which is cheaper than testing | 0 | 0 |
| 34 | 2 | Gaps in clinical knowledge and testing capabilities | Not identifying supplements as medications, and not reporting them | 0 | 0 |
| 35 | 2 | Patient limitations, financial barriers, and lack of resources | Patients can be unaware of exposures at work or at home, e.g., water damage | 0 | 0 |
| 36 | 2 | Patient limitations, financial barriers, and lack of resources | There are costs related to the after effects of inspection, such as lower resale value of the house, or inability to avoid work exposures or change jobs, patients may not want to even look for the antigen. | 0 | 0 |
| 37 | 2 | Patient limitations, financial barriers, and lack of resources | Veterans have service connection pressures - is the exposure work related or not? Often, we can't be definitive although there are system level pressures to be more definitive about workplace exposures. | 0 | 0 |
| 38 | 2 | There are many unknown and undiscovered antigens | Changes in the environment can change exposures or create new exposures. | 0 | 0 |
| Barriers discussed only in group 3 | | | | | |
|  | **Groups barrier was discussed in** | **Theme barrier was assigned to** | **Barrier** | **Number of Points**  **(n=150)** | **Number of Votes**  **(n=75)** |
| 39 | 3 | Problems with environmental inspections and testing | There is a lack of professional resources to look for antigens in the home or the workplace. | 8 | 4 |
| 40 | 3 | There are many unknown and undiscovered antigens | The ubiquitous nature for potential exposures e.g. mold in a significant number of ILD patients. | 5 | 2 |
| 41 | 3 | Problems with obtaining accurate and comprehensive exposure history | A really detailed exposure history is important but there are time constraints, patients do not complete questionnaire, and there is recall bias. | 3 | 1 |
| 42 | 3 | Gaps in clinical knowledge and testing capabilities | For serum precipitants, there are limited panels that do not cover all potential antigens that we do, and don't know. | 3 | 2 |
| 43 | 3 | Unclear Significance of Identified Exposures | They may have had the exposure for many years so they don't relate the exposure to their lung disease. | 2 | 1 |
| 44 | 3 | There are many unknown and undiscovered antigens | We don't know all the antigens that contribute to CHP. There are many undiscovered antigens | 1 | 1 |
| 45 | 3 | Problems with obtaining accurate and comprehensive exposure history | Physician awareness of the possible antigens - do I understand what certain antigens are well enough to explain them to patients, especially in laymen's terms? | 1 | 1 |
| 46 | 3 | Unclear Significance of Identified Exposures | There is difficulty in distinguishing between sensitization and disease - exposure-response relationship less clear than with acute HP, with serum test e.g. an agricultural community and multiple exposures. | 1 | 1 |
| 47 | 3 | Problems with obtaining accurate and comprehensive exposure history | Often the exposure could be hidden and it's difficult to identify the antigen immediately through targeted questioning. | 0 | 0 |
| 48 | 3 | Patient limitations, financial barriers, and lack of resources | Patients have a hard time recalling exposures - "recall bias" or simply poor memory | 0 | 0 |
| 49 | 3 | Unclear Significance of Identified Exposures | Identifying the past exposure that may not be ongoing and may or may be contributing to the development but maybe not progression of disease- results in challenges with confirming the diagnosis. | 0 | 0 |
| 50 | 3 | Problems with obtaining accurate and comprehensive exposure history | It is difficult trying to prompt patients to recall their exposure histories, particularly the temporal relationship, how far back should we ask? | 0 | 0 |
| 51 | 3 | Problems with environmental inspections and testing | Having tools for identifying home moisture and its influence in identifying antigens in the home - e.g. cardboard in the home. | 0 | 0 |
| 52 | 3 | Gaps in clinical knowledge and testing capabilities | There is diagnostic confusion for chronic UIP and IPF when making the diagnosis of CHP, many similarities, and overlap on path. Not a very clean diagnosis along with not being able to identify antigen e.g: concept of air trapping may be arbitrary | 0 | 0 |
| 53 | 3 | Patient limitations, financial barriers, and lack of resources | The cost and availability of home evaluations e.g. for mold is prohibitive for patients. Sometimes paying for a service that may not be accurate or give false results. | 0 | 0 |
| 54 | 3 | Gaps in clinical knowledge and testing capabilities | We are limited in our cultural knowledge of potential antigens e.g. a limited awareness of East Asian cohort home/work environment, other geographic regions of the US. | 0 | 0 |
| 55 | 3 | Gaps in clinical knowledge and testing capabilities | Training outside of a center that doesn't have an ILD center makes it difficult to pick up a CHP diagnosis – there is a need for greater provider education of HP. | 0 | 0 |
| 56 | 3 | Patient limitations, financial barriers, and lack of resources | The cost and general burden of asking a patient to change their environment or asking them to relocate | 0 | 0 |
| 57 | 3 | Gaps in clinical knowledge and testing capabilities | The difficulty of assessing withdrawal tests, how long should someone be away and determining if that makes the test positive. | 0 | 0 |
| 58 | 3 | Individual patient beliefs, emotions, and attachments to antigen source | Patients not wanting to disclose significant exposure histories. | 0 | 0 |
| 59 | 3 | Problems with obtaining accurate and comprehensive exposure history | A lack of knowledge or ability to access standardized workplace exposure evaluations specifically the NIOSH CDC HHE evaluation. | 0 | 0 |
| 60 | 3 | Problems with environmental inspections and testing | The validation of standardized moisture testing/assessment tool in CHP | 0 | 0 |

Key: In this table, individual barriers are organized by the group that it was identified in. This begins with barriers that were discussed in more than one group followed by the individual barriers that were discussed in either group 1,2, or 3 respectively. For each group, the barriers are in order of highest to lowest number of points.

**Table S2: List of all Individual Barriers to Antigen Avoidance**

|  | Group | Theme | Barrier | Points  (n=150) | Votes  (n=75) |
| --- | --- | --- | --- | --- | --- |
| All barriers discussed in more than 1 group | | | | | |
|  | **Groups barrier was discussed in** | **Theme barrier was assigned to** | **Barrier** | **Number of Points**  **(n=150)** | **Number of Votes**  **(n=75)** |
| 1 | 1,2,3 | Patient limitations, financial barriers, and lack of resources | The associated cost- including remediation of a home, moving to a different home, changing occupation, or livelihood | 53 | 21 |
| 2 | 1,3 | Effects on Employment | When there is an exposure at the workplace, an employer may be unable or unwilling to remediate or the livelihood is tied to the exposure and the patient is unable to leave their job. | 8 | 4 |
| 3 | 1,3 | Limitations with environmental remediation | Total or zero avoidance may not be possible. | 7 | 3 |
| 4 | 1 | Gaps in clinical knowledge and testing capabilities | Mis-identification of the correct antigen | 3 | 3 |
| 5 | 1,2,3 | Individual patient beliefs, emotions, and attachments to antigen source | The patient has a hobby, passion, or emotional connection to the exposure, or something associated with the exposure, this may affect quality of life. | 14 | 9 |
| 6 | 1,3 | Individual patient beliefs, emotions, and attachments to antigen source | Patients are unwilling to give up their pet (e.g. bird). | 2 | 1 |
| 7 | 1,3 | Patient imitations, financial barriers and lack of resources | Inability to avoid the antigen because of family members either who control the environment or who cannot move. | 1 | 1 |
| 8 | 1,2,3 | Individual patient beliefs, emotions, and attachments to antigen source | Patients do not believe the antigen identified is causing their disease | 6 | 4 |
| Barriers discussed only in group 1 | | | | | |
|  | **Groups barrier was discussed in** | **Theme barrier was assigned to** | **Barrier** | **Number of Points**  **(n=150)** | **Number of Votes**  **(n=75)** |
| 9 | 1 | Limitations with environmental remediation | It is difficult to alter the environment of public housing, often patients do not have control of this. | 2 | 1 |
| 10 | 1 | Patient imitations, financial barriers and lack of resources | Absence of insurance coverage, both health and homeowners. | 0 | 0 |
| 11 | 1 | Limitations with environmental remediation | Inability to avoid recurrent flooding of the home | 0 | 0 |
| 12 | 1 | Patient imitations, financial barriers and lack of resources | Patients’ income may decline because of the exposure, lack of ability to advance. | 0 | 0 |
| 13 | 1 | Individual patient beliefs, emotions, and attachments to antigen source | Patients do not want to wear protective equipment at job or hobby. | 0 | 0 |
| 14 | 1 | Limitations with environmental remediation | Seasonal variability in mold growth may cause a perception that a problem is fixed that is not fixed. | 0 | 0 |
| 15 | 1 | Gaps in clinical knowledge and testing capabilities | We may have perceived that an exposure is removed but the commercial tests are not sensitive enough to know if it is completely removed. | 0 | 0 |
| Barriers discussed only in group 2 | | | | | |
|  | **Groups barrier was discussed in** | **Theme barrier was assigned to** | **Barrier** | **Number of Points**  **(n=150)** | **Number of Votes**  **(n=75)** |
| 16 | 2 | Effects on Employment | Work exposure may lead to job switches, vocational rehab, or disability to avoid Antigen. | 11 | 5 |
| 17 | 2 | Gaps in clinical knowledge and testing capabilities | Lack of guidelines on how to remediate from an exposure | 10 | 6 |
| 18 | 2 | Patient imitations, financial barriers and lack of resources | Removal or avoidance may not be under patients' control. | 7 | 4 |
| 19 | 2 | Limitations with environmental remediation | Once environment is abated, exposure may still continue outside the abated environment. | 2 | 1 |
| 20 | 2 | Individual patient beliefs, emotions, and attachments to antigen source | Patients sometimes want to keep exposure to antigen even if it means harm. | 0 | 0 |
| 21 | 2 | Patient imitations, financial barriers and lack of resources | Due to cost, some patients are more affected by the disease than others who can afford to make changes - this can be emotionally difficult for the clinician. | 0 | 0 |
| 22 | 2 | Limitations with environmental remediation | Sometimes exposures can recur after remediation - e.g., mold recurrence. | 0 | 0 |
| Barriers discussed only in group 3 | | | | | |
|  | **Groups barrier was discussed in** | **Theme barrier was assigned to** | **Barrier** | **Number of Points**  **(n=150)** | **Number of Votes**  **(n=75)** |
| 23 | 3 | Individual patient beliefs, emotions, and attachments to antigen source | When there is a lack of clinical improvement despite antigen avoidance, this makes it hard to convince patient to avoid. | 11 | 5 |
| 24 | 3 | Gaps in clinical knowledge and testing capabilities | Lack of guidelines on what is acceptable or enough antigen avoidance. | 13 | 7 |
| 25 | 3 | Individual patient beliefs, emotions, and attachments to antigen source | There may be a necessity of the antigen. i.e.: down coat in cold weather. | 0 | 0 |
| 26 | 3 | Gaps in clinical knowledge and testing capabilities | Are the providers even right that the antigen is successfully identified? | 0 | 0 |
| 27 | 3 | Individual patient beliefs, emotions, and attachments to antigen source | It is difficult to have and to know that there is deliberate and complete avoidance of antigen from patient and family members. | 0 | 0 |
| 28 | 3 | Limitations with environmental remediation | There is difficulty avoiding multiple antigens. Avoiding one is difficult enough but avoiding multiple is even more difficult | 0 | 0 |

Key: In this table, individual barriers are organized by the group that it was identified in. This begins with barriers that were discussed in more than one group followed by the individual barriers that were discussed in either group 1,2, or 3 respectively. For each group, the barriers are in order of highest to lowest number of points.
